# Will the NHS continue to function in an influenza pandemic? a survey of healthcare workers in the West Midlands, UK

**DOI:** 10.1186/1471-2458-9-142

**Published:** 2009-05-14

**Authors:** Sarah Damery, Sue Wilson, Heather Draper, Christine Gratus, Sheila Greenfield, Jonathan Ives, Jayne Parry, Judith Petts, Tom Sorell

**Affiliations:** 1 Department of Primary Care Clinical Sciences, University of Birmingham, Edgbaston, Birmingham, B15 2TT, UK; 2 Department of Public and Occupational Health, Department of Primary Care Clinical Sciences, University of Birmingham, Edgbaston, Birmingham, B15 2TT, UK; 3 School of Geography, Earth and Environmental Sciences, University of Birmingham, Edgbaston, Birmingham, B15 2TT, UK; 4 Centre for the Study of Global Ethics, University of Birmingham, Edgbaston, Birmingham, B15 2TT, UK

## Abstract

**Background:**

If UK healthcare services are to respond effectively to pandemic influenza, levels of absenteeism amongst healthcare workers (HCWs) must be minimised. Current estimates of the likelihood that HCWs will continue to attend work during a pandemic are subject to scientific and predictive uncertainty, yet an informed evidence base is needed if contingency plans addressing the issues of HCW absenteeism are to be prepared.

**Methods:**

This paper reports the findings of a self-completed survey of randomly selected HCWs across three purposively sampled healthcare trusts in the West Midlands. The survey aimed to identify the factors positively or negatively associated with willingness to work during an influenza pandemic, and to evaluate the acceptability of potential interventions or changes to working practice to promote the continued presence at work of those otherwise unwilling or unable to attend. 'Likelihood' and 'persuadability' scores were calculated for each respondent according to indications of whether or not they were likely to work under different circumstances. Binary logistic regression was used to compute bivariate and multivariate odds ratios to evaluate the association of demographic variables and other respondent characteristics with the self-described likelihood of reporting to work.

**Results:**

The survey response rate was 34.4% (n = 1032). Results suggest absenteeism may be as high as 85% at any point during a pandemic, with potential absence particularly concentrated amongst nursing and ancillary workers (OR 0.3; 95% CI 0.1 to 0.7 and 0.5; 95% CI 0.2 to 0.9 respectively).

**Conclusion:**

Levels of absenteeism amongst HCWs may be considerably higher than official estimates, with potential absence concentrated amongst certain groups of employees. Although interventions designed to minimise absenteeism should target HCWs with a low stated likelihood of working, members of these groups may also be the least receptive to such interventions. Changes to working conditions which reduce barriers to the *ability *to work may not address barriers linked to *willingness *to work, and may fail to overcome HCWs' reluctance to work in the face of what may still be deemed unacceptable risk to self and/or family.

## Background

Healthcare workers (HCWs) will be at the forefront of response to pandemic influenza, and if healthcare services are to be provided at levels appropriate to address the additional demands and pressures that a pandemic will place upon them, absenteeism from work amongst HCWs must be minimised. Limited research has been conducted into establishing the likely levels of absenteeism amongst HCWs during an influenza pandemic, with findings varying significantly in both estimates of the likelihood that HCWs of different kinds would report to work, and in assessments of factors that may have a significant influence on decision-making.

For instance, research amongst primary care physicians in Singapore suggests potential levels of absenteeism (in the face of Avian influenza) as low as 11.8% due to strongly held perceptions of a professional obligation to work despite personal risks [1] This finding is corroborated by research focusing on general practitioners in Australia [2]. In contrast, in a US-based survey, Balicer *et al *[3] found that nearly half of healthcare employees may fail to report for duty during an influenza pandemic, with potential absenteeism particularly high amongst technical and support staff. Ehrenstein *et al *[4] report that 28% of German HCWs may remain absent from work in order to protect themselves and their families, factors also found to be significant by Qureshi *et al *in a US survey of HCWs' likely response to catastrophic events [5].

The World Health Organization (WHO) has recommended national pandemic influenza preparedness plans are produced [6], and pandemic influenza has recently been identified by the UK Government as the greatest threat to national security in terms of likelihood of occurrence and potential severity of impact [7]. Estimates of the likely severity of an influenza pandemic in the UK, and the corresponding levels of absenteeism that might be expected amongst HCWs, have been continually revised. Initial estimates assumed absenteeism to be around twice that of normal National Health Service (NHS) levels, which are typically between 4–5% [8]. More recent modelling has predicted between 30–35% absenteeism at the peak of a pandemic, based on cumulative effects of staff illness and the possible closure of schools/childcare services [9]. Recent human resources guidelines issued by the UK Department of Health (DH) increase estimates further, suggesting that up to 50% of HCWs may require time off at the peak of a pandemic [10].

However, estimates underlying current preparedness plans are subject to considerable scientific and predictive uncertainty, and with the potentially limited domestic applicability of studies conducted internationally, there is a need for informed research which helps establish an evidence base about UK HCWs' willingness to work during an influenza pandemic. This paper presents the findings of a large scale survey of HCWs in the West Midlands which aimed to investigate the factors associated with willingness to work during an influenza pandemic, and to identify changes to working practice which may promote the continued presence at work of those HCWs otherwise unwilling or unable to attend.

## Methods

This survey is the second part of a two-phase multi-method study [11] conducted in the West Midlands, UK. The target population included all categories of HCWs (e.g. hospital doctors, nursing staff, professions allied to medicine (PAM) (e.g. pharmacists, radiographers, phlebotomists), healthcare managers, ancillary staff (e.g. porters, hotel services, mortuary attendants), GPs and community HCWs). Survey recipients were randomly selected from staff databases provided by Human Resources (HR) contacts, in each of three participating Trusts: one acute teaching, one rural district general, and one Primary Care Trust (PCT). Trusts were purposively recruited to ensure responses from all types of HCWs across a wide demographic range, and across a range of healthcare settings, and sampling was stratified by Trust. Self-completion surveys were sent to a total of 3,000 HCWs between July and September 2008. This mailing assumed a conservative 25% response rate, to yield a sample size sufficient to determine the overall proportion of respondents with a positive attitude to continuing to work during a pandemic with a precision of 2% (95% confidence), based on a worst case assumption of 50% reporting a positive attitude.

Mailing addresses of current staff were obtained from Human Resources (HR) departments in one NHS Trust and the PCT. Questionnaires were mailed directly to individuals at their work address. In the remaining NHS Trust, surveys were distributed internally to staff in the relevant HCW categories by clinical managers. Recipients wishing to participate in the survey were given the choice of completing a paper version, returned via Freepost envelope directly to the University of Birmingham or submitting their responses online. Non-respondents received one reminder (mailed in September 2008). No incentive was offered for survey completion.

### Survey content

Survey content was informed by the findings of qualitative research conducted in phase one of the study, as discussed by Ives *et al*. [12]. The survey included closed questions (see Additional file 1 for survey instrument) on demographic characteristics; HCW employment category; nature of employment; home circumstances and caring responsibilities; the distance from home to work, and the mode of transport typically used to travel to and from work. The survey also included questions eliciting agreement or disagreement with various ethical principles, and respondents' perceptions of the impact of pandemic influenza, the perceived likely incidence of infection and mortality, and the demographic groups perceived to have the highest morbidity and mortality during a pandemic.

In order to investigate the likelihood that an individual would continue to work during pandemic influenza, one question (Q4) outlined a number of circumstances (n = 12) which may arise during a pandemic (e.g. infection of self and/or family members, disruption to childcare etc.). Respondents indicated the likelihood of their continuing to work under each circumstance ('likely', 'unlikely', 'don't know' or 'not applicable'). A further question (Q5) was designed to identify the acceptability of various incentives, which may persuade otherwise reluctant individuals to continue working during a pandemic (e.g. the option to work more flexible hours, provision of childcare etc.). Respondents indicated for each intervention (n = 12) whether it might make them 'more likely' to work, or 'about the same'.

### Data analysis

**Figure 1:**
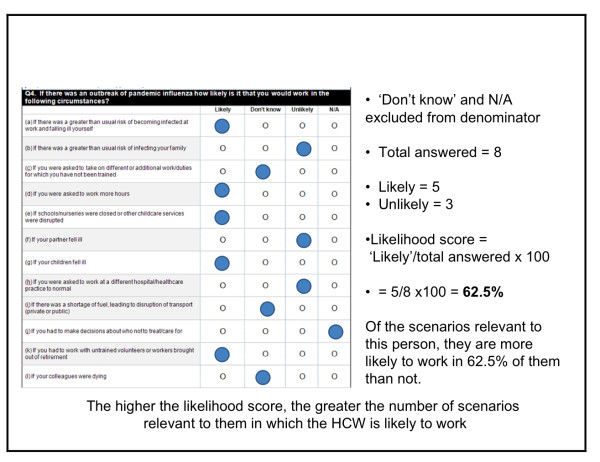
**Worked example of calculation of 'likelihood' score**.

Analysis focused on the characteristics of respondents giving positive or negative ratings to their perceived likelihood of working under different circumstances, and on the characteristics of those indicating whether a given intervention would make them more likely to continue working. A 'likelihood' score was calculated for each individual, based on the percentage of circumstances relevant to them in which they indicated that they would be 'likely' to work. The higher the score, the greater the proportion of circumstances in which a HCW might work. Similarly, a 'persuadability' score was developed, derived from the percentage of interventions that an individual indicated may make them 'more likely' to continue working during a pandemic. In calculating both 'likelihood' and 'persuadability' scores, 'don't know' and/or 'not applicable' responses were excluded (see Figure 1 for a sample calculation).

Responses were dichotomised between those with a 'likelihood' score of 100%, and those scoring <100%. Binary logistic regression was used to compute bivariate and multivariate Odds Ratios to evaluate the association of demographic variables and other respondent characteristics with the self-described likelihood of reporting to work. The multivariate regression model included adjustment for age, gender, job category, living arrangements, type of work (part-time/full-time) and caring responsibilities. As it might be expected that policy interventions to reduce absenteeism during a pandemic should be targeted towards those who may be less willing or less able to work, those constituting the <100% 'likelihood' group were isolated, and binary logistic regression was again used to calculate bivariate and multivariate Odds Ratios associating 'persuadability' scores for these respondents with demographic and employment characteristics. For both 'likelihood' and 'persuadability', logistic regression was used to identify the key determinants of agreement/disagreement with individual circumstances/policy interventions. In order to assess non-response bias, age, gender, and job category distributions for respondents and non-respondents were tested using chi-square analysis. All data were analysed using SPSS (version 15.0).

### Ethical Approval

National Research Ethics Service (NRES) approval for this project was granted by Nottingham Research Ethics Committee 2 (Ref: 07/H0408/120), and R&D approval was gained from each participating trust.

## Results

Of the 3,000 questionnaires distributed, 1,032 (34.4%) were returned complete, and 70 were returned blank, indicating a desire not to receive a reminder questionnaire. No statistically significant difference in age, gender or job category was found between respondents and non-respondents (Table 1), suggesting that the responses received reflected the demographic composition of the wider survey population. Response rates varied by occupational group, ranging from 23.5% from GPs (n = 141) to 50.3% from managers (n = 151).

**Table 1:** Survey response rates by occupational category and analysis of non-response bias (age, gender)

**Job category**	**Surveys sent**	**Number returned**	**Response rate (%)**	Non-response bias, gender*	**Non-response bias, age group**
Doctors	500	122	24.4	X^2 ^= 0.03; p = 0.863	X^2 ^= 4.38; p = 0.223
Nurses	300	134	44.7	X^2 ^= 0.03; p = 0.863	X^2 ^= 6.13; p = 0.106
Professions Allied to Medicine (PAM)	300	149	49.7	X^2 ^= 0.32; p = 0.572	X^2 ^= 6.29; p = 0.098
Ancillary	500	179	35.8	X^2 ^= 1.09; p = 0.297	X^2 ^= 4.04; p = 0.257
Manager	300	151	50.3	X^2 ^= 0.02; p = 0.888	X^2 ^= 4.82; p = 0.186
GP	600	141	23.5	No data	No data
Community HCW	500	156	31.2	X^2 ^= 0.49; p = 0.484	X^2 ^= 3.77; p = 0.287
					
All respondents	3000	1032	34.4	X^2 ^= 1.98; p = 0.159	X^2 ^= 3.77; p = 0.287

* Information on the gender and age group of survey recipients was not provided on the database of GPs supplied by the PCT

The study population (Table 2) comprised more females than males (ratio 2.18:1), and 57.9% of survey respondents were aged 41 or above (n = 597). The largest employment category was ancillary workers, who constituted 17.3% of respondents (n = 179); the smallest was doctors (11.8%; n = 122). HCWs who indicated some form of caring responsibility (for children aged under 16 and/or elderly dependents) constituted just over half of responses received (50.2%; n = 518), and full-time workers outnumbered part-time workers (ratio 3:1; n = 767 vs. n = 260).

**Table 2:** Demographic characteristics of the study population and likelihood of reporting to work

**Characteristic**	n(%)*	**Mean likelihood of working score**	**Bivariate OR (95% CI)**	**Multivariate OR (95% CI) §**
**Age**				
16–30	180 (17.5)	57.2	Reference	Reference
31–40	253 (24.6)	51.8	0.5 (0.2 – 0.9)	0.7 (0.3 – 1.4)
41–50	331 (32.1)	61.1	1.5 (0.9 – 2.6)	2.3 (1.2 – 4.4)
51+	266 (25.8)	64.7	1.8 (1.0 – 3.0)	1.8 (0.9 – 3.5)
				
**Gender**				
Male	323 (31.5)	64.7	Reference	Reference
Female	704 (68.5)	56.4	0.6 (0.4 – 0.9)	1.0 (0.7 – 1.6)
				
**Job Category**				
Doctor	122 (11.8)	67.0	Reference	Reference
Nurse	134 (13.0)	49.3	0.3 (0.1 – 0.7)	0.3 (0.1 – 0.8)
Professions Allied to Medicine (PAM)	149 (14.4)	60.7	1.0 (0.5 – 1.8)	1.0 (0.5 – 1.9)
Ancillary	179 (17.3)	49.0	0.5 (0.2 – 0.9)	0.5 (0.2 – 0.9)
Manager	151 (14.6)	63.5	1.0 (0.6 – 1.9)	0.8 (0.4 – 1.6)
GP	141 (13.7)	71.4	1.3 (0.7 – 2.4)	1.4 (0.7 – 2.8)
Community HCW	156 (15.1)	55.7	0.5 (0.2 – 0.9)	0.5 (0.2 – 0.9)
				
**Living Arrangements**				
Live with children under 16	442 (43.1)	53.7	Reference	Reference
No children under 16	389 (38.0)	64.5	2.6 (1.7 – 3.9)	1.9 (0.9 – 4.3)
Live alone	101 (9.9)	69.2	4.4 (2.6 – 7.6)	3.7 (1.6 – 9.0)
Share with friends	28 (2.7)	64.5	4.4 (1.8 – 10.6)	4.7 (1.5 – 15.5)
With parents/relatives	65 (6.3)	47.8	0.2 (0.1 – 1.3)	0.2 (0.1 – 1.5)
				
**Caring Role(s)**				
No children under 16 or elderly dependents	518 (50.2)	63.7	Reference	Reference
Children <16 and/or elderly dependents	514 (49.8)	54.4	0.4 (0.3 – 0.6)	0.8 (0.4 – 1.7)
				
**Nature of Employment**				
Full Time	767 (74.7)	62.0	Reference	Reference
Part Time	260 (25.3)	50.5	0.4 (0.2 – 0.6)	0.4 (0.3 – 0.8)
				
**All Respondents**	1032 (100%)	59.3		

* Percentages may not total 100 due to missing responses

§ Adjusted for Age, Gender, Job Category, Living Arrangements, Caring Role(s) and Nature of Employment

### Likelihood of working

**Figure 2:**
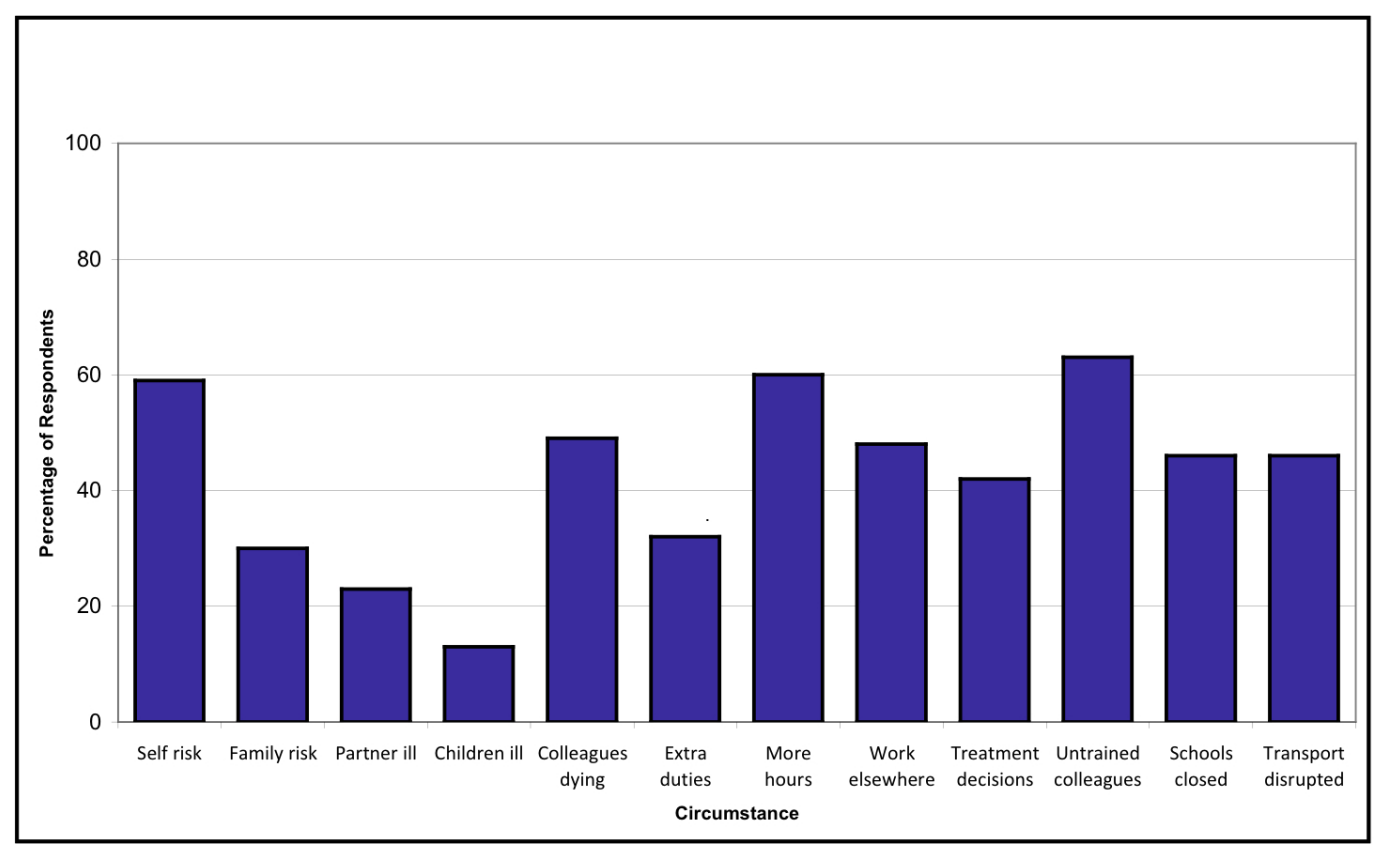
**Proportion of HCWs who indicated they would be 'likely' to work in a given circumstance**.

Figure 2 shows the proportion of respondents who indicated that they would be 'likely' to work in a given circumstance. Factors with the greatest potential impact on likelihood of working were illness to children (13% of respondents would continue to work in this circumstance; n = 134) and illness to partner (23%; n = 237). The potential need to work more hours than normal (60%; n = 619) or working with untrained volunteers (63%; n = 650) were reported to have the lowest potential impact on likelihood of working.

The mean likelihood score (i.e. the mean percentage of circumstances which may arise during a pandemic under which individuals stated they would be willing to work) for all respondents was 59.3%. Only 149 respondents (14.4%) indicated that they would be likely to work in all (individually relevant) circumstances (a likelihood score of 100%) (Table 3). Females were significantly less likely to work during a pandemic than males (bivariate OR: 0.6; CI 0.4 to 0.9), as were part-time employees compared to full-time workers (bivariate OR: 0.4; CI 0.2 to 0.6), and HCWs with caring responsibilities compared to those without children or elderly dependents (bivariate OR: 0.4; CI 0.3 to 0.6). Across employment categories, nurses, ancillary workers and community HCWs had the lowest reported likelihood of working (bivariate OR: 0.3; CI 0.1 to 0.7; 0.5; CI 0.2 to 0.9 and 0.5; CI 0.2 to 0.9 respectively).

**Table 3:** The number of individually relevant circumstances in which respondents stated they would be likely to work

**Characteristic**	0*	**1 to 3**	**4 to 6**	**7 to 9**	**10 or 11**	**12**	**Total**
**Age**							
							
16–30	12.8 11.1	7.2	11.7	31.1	25.0	12.2	100
31–40		7.5	22.9	31.6	20.9	5.9	100
41–50	8.5	8.2	13.3	26.9	25.7	17.5	100
51+	6.8	6.8	10.9	30.5	25.2	19.9	100
							
							
**Gender**							
							
Male	9.0	5.9	9.9	25.1	31.6	18.6	100
Female	9.7	8.2	17.0	31.7	20.7	12.6	100
							
							
**Job Category**							
							
Doctors	5.7	5.7	10.7	22.1	37.7	18.0	100
Nurses	14.9	14.2	14.2	29.1	21.6	6.0	100
PAM	7.4	7.4	14.8	31.5	20.8	18.1	100
Ancillary	16.2	8.9	20.7	28.5	16.2	9.5	100
Managers	4.6	7.3	14.6	30.5	24.5	18.5	100
GPs	4.3	3.5	9.2	26.2	34.0	22.7	100
Community HCW	11.5	5.1	16.7	37.8	19.2	9.6	100
							
							
**Caring Role(s)**							
							
No children under 16 or elderly dependents	8.5	6.0	10.8	28.2	27.2	19.3	100
Children <16 and/or elderly dependents	10.5	8.9	18.7	31.1	21.2	9.5	100
							
							
**Nature of Employment**							
							
Full time	8.7	6.8	12.0	29.1	26.6	16.8	100
Part time	11.9	9.6	22.3	31.2	17.7	7.3	100
							
							
**All Respondents**	9.5	7.5	14.7	29.7	24.2	14.4	100

*Figures relate to the percentage of respondents in each demographic or employment category

Multivariate analysis showed that those who lived alone or who shared with friends were significantly more likely to report to work than those in households with children (multivariate OR: 3.7; CI 1.6 to 9.0 and 4.7; CI 1.5 to 15.5 respectively). GPs indicated a higher likelihood of working during a pandemic than other categories of HCW, along with respondents aged 41–50 years (multivariate OR 2.3; 1.2 to 4.4).

### Predictors of reported likelihood of working under different circumstances

Table 4 shows the demographic and employment characteristics associated with whether an individual would be potentially 'likely' or 'unlikely' to work under a given scenario. Infection of self and/or family was most strongly associated with different respondent characteristics. Gender was a significant predictor of the likelihood of working if children fell ill: females were significantly less likely to continue to work under this circumstance than males (OR 0.4; CI 0.3 to 0.7). Work type was also a significant predictor of likelihood of working under this scenario, with part-time workers less likely to work than full-time HCWs (OR 0.4; CI 0.2 to 07).

**Table 4:** Predictors of likelihood of working under different circumstances

**Circumstances:** "How likely is it that you would work if... ?" * §	**Gender**	**Job Type**	**Carer**	**Work Type (FT/PT)**	**Age Group**	**Living Arrangements**
There was a greater than usual risk of becoming infected at work and falling ill yourself	0.454	**<0.001**	0.106	0.129	0.005	0.812
There was a greater than usual risk of infecting your family	0.289	**<0.001**	0.379	0.004	**<0.001**	0.274
Your partner fell ill	0.056	**<0.001**	0.185	0.726	0.074	0.037
Your children fell ill	**<0.001**	0.022	0.325	**0.002**	0.108	0.912
Schools/nurseries were closed or childcare services disrupted	0.038	0.365	0.336	**<0.001**	**0.002**	0.043
You were asked to take on duties for which you have not been trained	0.691	**<0.001**	0.448	0.572	0.267	0.029
You were asked to work more hours	0.997	0.077	0.100	0.015	0.014	0.461
You were asked to work at a different site to normal	0.757	**<0.001**	0.340	0.038	0.102	0.061
You had to decide who not to treat/care for	0.005	**<0.001**	0.067	0.445	0.751	0.012
You had to work with untrained volunteers or workers brought out of retirement	0.932	**<0.001**	0.961	0.836	0.470	0.282
There was a shortage of fuel leading to disruption of transport	0.278	0.200	0.541	0.203	0.402	0.182
Your colleagues were dying	0.189	**<0.001**	0.497	0.695	**0.002**	0.625

* P values: Bonferroni correction factor applied to account for multiple testing: cut-off for significance adjusted to 0.003

§ Regression model adjusted for Gender, Job Type, Caring Role(s), Work Type, Age Group and Living Arrangements

In the case of personal infection risk, doctors and GPs were the most likely to continue working despite the risk, compared with HCWs in other job categories (OR: 2.6; CI 1.3 to 5.4 for doctors, and OR: 4.8; CI 2.2 to 10.4 for GPs). Similarly, GPs were also significantly more likely to work despite the risk of infecting family members (OR: 3.8; CI 2.1 to 7.0) or if a partner fell ill (OR: 3.0; CI 1.6 to 5.6).

For all circumstances relating to working conditions rather than personal or family risk, job type was the only significant predictor of whether an individual was likely to agree with the survey statements. Compared to those in other HCW categories, nurses were particularly reluctant to work if they had to take on duties for which they had not received training (OR 0.5; CI 0.3 to 0.9). They were similarly unlikely to attend if asked to work at a different site to normal (OR 0.2; CI 0.1 to 0.3), as were ancillary workers (OR: 0.3; CI 0.2 to 0.5). Working with untrained volunteers or those brought out of retirement produced a mixed response on the basis of job type – nurses and ancillary workers would be reluctant to work under this scenario (OR: 0.5; CI 0.2 to 0.9 and OR: 0.5; CI 0.3 to 0.9 respectively), whereas GPs would be particularly likely to continue working on this basis (OR: 4.0; CI 1.4 to 11.9).

### 'Persuadability' of those with a <100% reported likelihood of working

Isolating those respondents who reported a lower probability of working on the basis of responses to the 'likelihood' circumstances (n = 883), Table 5 shows how 'persuadable' these respondents may be towards overcoming their unwillingness or inability to work if changes to working conditions were introduced during a pandemic. The mean 'persuadability' score for the selected respondents was 69.91% (i.e. that for the suggested policy interventions relevant to them, nearly 70% of these interventions would persuade HCWs to continue working). Demographic groups with the highest persuadability scores were those in the 16–30 age group (n = 158; mean score 75.24%); community HCWs (n = 141; mean score 75.49%); HCWs living in households without children (n = 418; mean score 71.84%), and those living with parents or relatives (n = 64; mean score 74.23%). Groups with the lowest mean persuadability scores were nurses (n = 126; mean score 66.23%) and those living with friends (n = 20; mean score 54.96%).

**Table 5:** 'Persuadability' score by demographic characteristics, and likelihood of being 'persuaded' to work in an influenza pandemic.

**Characteristic**	n (%)*	**Mean persuadability score**	**Bivariate OR (95% CI)**	**Multivariate OR (95% CI)§**
**Age**				
16–30	158 (17.9)	75.24	Reference	Reference
31–40	238 (27.0)	71.13	0.8 (0.5 – 1.3)	0.9 (0.5 – 1.7)
41–50	273 (30.9)	68.93	0.7 (0.4 – 1.1)	0.7 (0.4 – 1.3)
51+	213 (24.1)	65.76	0.8 (0.5 – 1.4)	0.9 (0.5 – 1.7)
				
**Gender**				
Male	263 (29.8)	66.87	Reference	Reference
Female	615 (69.6)	71.27	1.3 (0.8 – 1.9)	1.3 (0.8 – 2.1)
				
**Job Category**				
Doctor	100 (11.3)	73.01	Reference	Reference
Nurse	126 (14.3)	66.23	1.0 (0.5 – 2.0)	0.9 (0.4 – 2.0)
PAM	122 (13.8)	68.79	0.5 (0.2 – 1.2)	0.5 (0.2 – 1.1)
Ancillary	162 (18.3)	68.88	1.3 (0.7 – 2.6)	1.2 (0.6 – 2.5)
Manager	123 (13.9)	67.42	0.8 (0.4 – 1.7)	0.8 (0.4 – 1.7)
GP	109 (12.3)	69.71	0.7 (0.3 – 1.5)	0.9 (0.4 – 1.9)
Community HCW	141 (16.0)	75.49	1.7 (0.9 – 3.2)	1.4 (0.7 – 2.9)
				
**Living Arrangements**				
Live with children under 16	405 (45.9)	68.74	Reference	Reference
No children under 16	315 (35.7)	71.84	1.4 (0.9 – 2.0)	2.2 (1.1 – 4.3)
Live alone	72 (8.2)	68.26	1.3 (0.7 – 2.5)	1.9 – (0.8 – 4.5)
Share with friends	20 (2.3)	54.96	1.0 (0.3 – 3.6)	1.5 (0.4 – 6.3)
With parents/relatives	64 (7.2)	74.23	2.0 (1.0 – 3.7)	2.6 (1.7 – 6.4)
				
**Caring Role(s)**				
No children under 16 or elderly dependents	418 (47.3)	70.35	Reference	Reference
Children <16 and/or elderly dependents	465 (52.7)	69.51	0.9 (0.6 – 1.3)	0.5 (0.3 – 0.9)
				
**Nature of Employment**				
Full Time	638 (72.3)	70.34	Reference	Reference
Part Time	241 (27.3)	68.83	0.7 (0.5 – 1.1)	0.7 (0.4 – 1.1)
				
**All Respondents**	883 (100%)	69.91		

Mean 'persuadability' score by demographic characteristics, and likelihood of being 'persuaded' to work (amongst respondents with <100% likelihood of working)* Percentages may not total 100 due to missing responses

§ Adjusted for Age, Gender, Job Category, Living Arrangements, Caring Role(s) and Nature of Employment

Few of the bivariate or multivariate Odds Ratios were significant at the 0.05 level. Those that did show significant results related to households without children being over twice as persuadable to work than households with children (multivariate OR: 2.2; CI 1.1 to 4.3), and HCWs with caring responsibilities for children and/or elderly dependents reported particularly low levels of persuadability in comparison to those without dependents (multivariate OR: 0.5; CI 0.3 to 0.9).

### Acceptability of policy interventions to increase attendance at work

**Figure 3:**
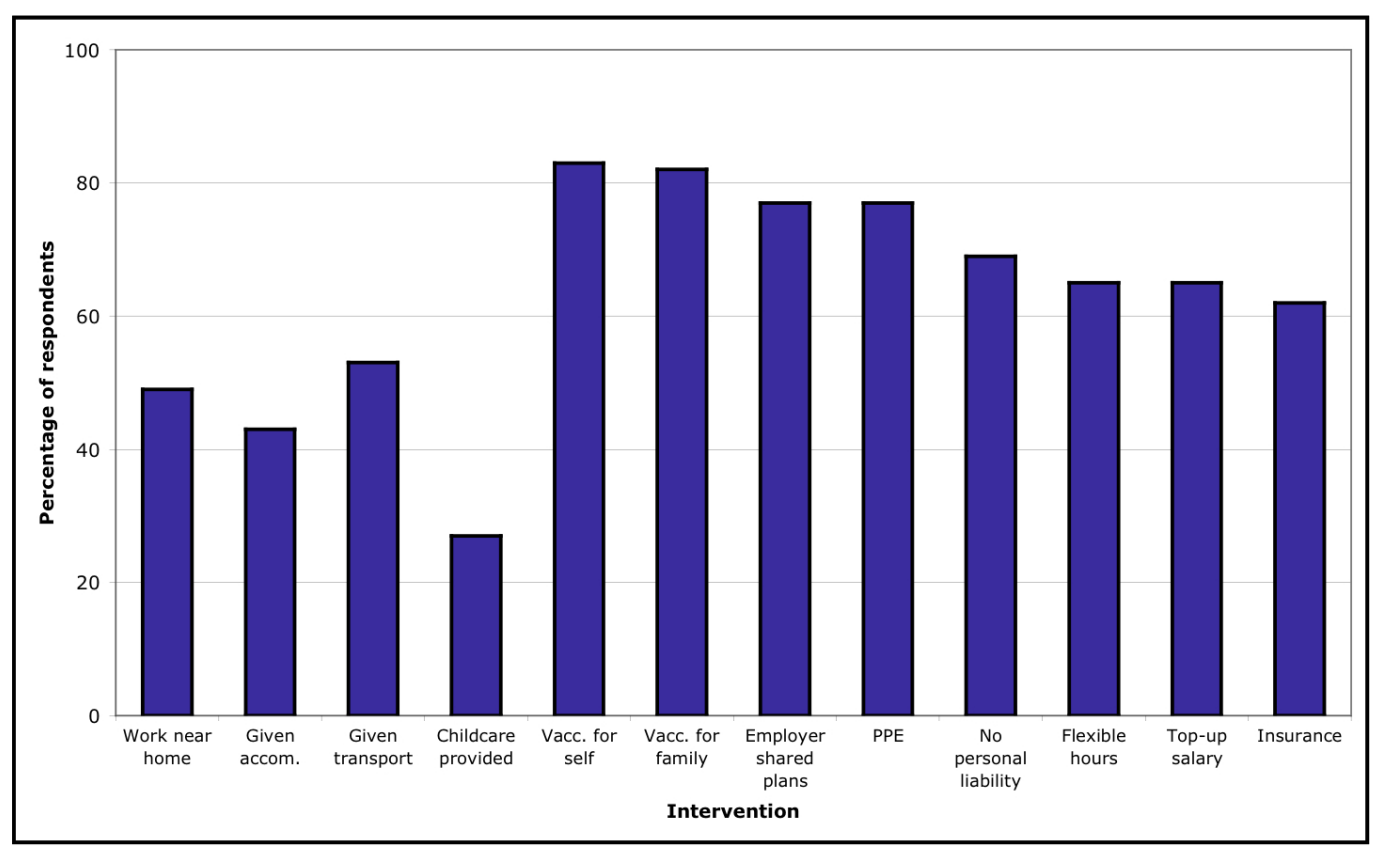
**Proportion of HCWs who indicated an intervention would make them 'more likely' to work**.

The potential impact of each of the suggested changes to working conditions in increasing HCWs' likely attendance at work varied considerably (Figure 3). Of the scenarios relevant to the 883 respondents identified as having a lower initial likelihood of reporting to work during a pandemic, the least influential interventions were shown to be the possibility of working nearer to home (50.1% agreed, n = 443); the provision of accommodation (43.1%, n = 381); the provision of transport (54.6%, n = 482), and the provision of childcare (60.8%, n = 261). The most influential interventions were the provision of vaccination for oneself and one's family (83.4%, n = 736 and 83.1%, n = 734 respectively).

These were followed by the provision of personal protective equipment (PPE) (77.8%, n = 687) and having employers share emergency plans with their employees (77.2%, n = 682). Interventions which would provide incentives or employee safeguards were also recognised as potentially beneficial: having employers accept liability for any mistakes made (68.6%, n = 589); being able to work flexible hours (66.7%, n = 589), receiving a top-up salary commensurate with the level of extra duties an individual may be asked to perform (66.8%, n = 590), and the provision of life/disability insurance (62.9%, n = 555).

Few overriding demographic or employment characteristics emerged as predictors of agreement or disagreement with specific policy interventions outlined in the survey (Table 6). Of the 12 potential interventions investigated, none would have encouraged all HCWs within a given demographic or employment group to work. In the case of the provision of facilities to prevent infection spread, significant associations were most often found within groups who indicated a low potential acceptance of such measures. For example, ancillary workers were significantly less likely to view the possibility of working at a site nearer to their homes as acceptable in comparison to other categories of HCW (OR 0.6; CI 0.3 to 0.9). Similarly, whilst those without caring responsibilities and HCWs who lived with parents indicated a significant increase in their likelihood of working if transport was provided (OR 2.2; CI 1.1 to 4.2 and OR 2.6; CI 1.1 to 6.4 respectively), nurses in particular were identified as being significantly less likely to respond positively to this intervention when compared to other job groups (OR 0.5; CI 0.3 to 0.8).

**Table 6:** Predictors of the influence of policy interventions on HCWs' potential decisions about working

**Interventions:** "Would you be more likely to work if... ?" * §	**Gender**	**Job Type**	**Carer**	**Work Type (FT/PT)**	**Age Group**	**Living Arrangement**
You were allowed to work at the nearest site to your home	0.059	0.187	0.521	0.710	0.880	0.086
You were provided with accommodation so that you do not take infection home	0.304	0.515	0.104	0.136	0.313	0.377
Your employer provided transport to get you to work and home again	0.007	0.022	0.158	0.310	0.156	0.010
Childcare was provided for you	0.188	0.127	0.755	0.975	0.013	0.699
You were offered vaccination (if available) and/or treatment if you fell ill	0.732	0.250	0.616	0.252	0.042	0.017
Your family were offered vaccination (if available) and/or treatment if they fell ill	0.993	0.709	0.302	0.400	0.060	**<0.001**
Your employer shared their emergency plans with you and told you in advance what would be expected of you during a pandemic	**0.002**	0.916	0.916	0.605	0.301	0.097
You were offered PPE when working with affected patients	0.053	0.465	0.585	0.379	0.425	0.029
Your employers accepted liability for any mistake made whilst doing a job you are not trained for	0.446	**0.002**	0.216	0.497	0.005	0.050
You were allowed to work more flexible hours	**0.002**	0.009	0.044	0.711	0.007	0.025
You were paid a top-up salary that reflected the level of duties you were asked to take on during a pandemic	0.105	0.093	0.145	0.987	**<0.001**	0.167
You were provided with life/disability insurance	0.450	0.090	0.359	0.333	0.014	0.152

* P values: Bonferroni correction factor applied to account for multiple testing: cut-off for significance adjusted to 0.003

§ Regression model adjusted for Gender, Job Type, Caring Role(s), Work Type, Age Group and Living Arrangements

Nurses were also significantly less likely to choose to work (compared to other types of HCW) if their employers accepted liability for mistakes (OR 0.3; CI 0.2 to 0.6), if they were offered more flexible working hours (OR 0.4; CI 0.2 to 0.7) or were paid a top-up salary (OR 0.4; CI 0.2 to 0.8). Flexibility of working hours was noted as potentially beneficial to respondents without children in comparison to those with children (OR 2.1; CI 1.0 to 4.4 and OR 0.5; CI 0.2 to 0.9 respectively). The interventions with the most significant positive associations were those where employers would offer some form of incentive to work during a pandemic.

## Discussion

An influenza pandemic will undoubtedly place the NHS under severe strain [6,7]. Although the potential severity and impact of a pandemic cannot be predicted, it is vital that contingency plans are prepared so that critical healthcare services, and above all patient care, can be maintained at appropriate levels both during the initial surge of a pandemic wave and beyond [13]. In preparing these contingency plans, informed estimates of the likely levels of absenteeism amongst front line HCWs are essential, as is an understanding of the factors which might contribute to an individual's decision to work (or not), and crucially, the interventions or changes to working conditions which may be most likely to influence otherwise reluctant HCWs to continue working.

The findings from this survey raise a number of key issues. First, potential levels of absenteeism amongst HCWs during a pandemic may be significantly higher than even the 50% worst-case scenario predicted by the DH [10]. Of all respondents, (n = 1032), only 149 individuals (14.4%) indicated that they would be likely to work during all of the potentially adverse circumstances identified in the survey which were relevant to them. This puts potential absenteeism at any one time during a pandemic as high as 85.6%.

Second, the self-reported likelihood of HCWs working during a pandemic shows considerable variation both within and across demographic and employment categories. Not only could absenteeism across all HCW categories be around 85%, but certain job types could be affected by higher levels of absenteeism than others. Whilst doctors and GPs reported a high likelihood of working (mean likelihood scores of 67% and 71.4% respectively), nurses, ancillary workers and community HCWs all expressed low potential levels of attendance at work during a pandemic (mean likelihood scores of 49.3%, 49% and 55.7%). It has been argued that clinical staff, and doctors in particular, may have a strong sense of professional obligation and an ethical duty to work, even in the face of high levels of personal risk [14-16]. Such factors may not be enough to ensure that nurses remain at work during a pandemic. Similarly, perceived obligations may have little impact on reducing absenteeism amongst ancillary workers [12].

In addition, perceived obligation to family members and caring for children and/or elderly dependents may compound problems associated with potentially high levels of absenteeism amongst some categories of HCWs. The survey demonstrated that those who work part-time, and those who care for dependents may be particularly liable to decide not to work during a pandemic in comparison to full-time workers and individuals without caring responsibilities (bivariate OR 0.4; CI 0.2 to 0.6 and 0.4; CI 0.3 to 0.6 respectively). Indeed, the analysis of response to the individual circumstances outlined by the survey clearly showed that the risks of infection to self or family, and illness of partner or children may be the deciding factor in many HCWs' decisions about working during a pandemic.

Given that potential levels of absenteeism may be significantly higher than current official estimates, and that absenteeism could be particularly marked amongst certain groups of HCW, official attempts to overcome the stated barriers to attendance at work are very important. Whilst the issues surrounding likelihood of working during a pandemic were relatively clear amongst survey respondents, the likely acceptability of certain interventions or changes to working conditions amongst those initially more reluctant to work is more difficult to quantify.

It is clear that beneficial interventions should be targeted towards those demographic or employment groups who are initially less likely to work, and who may constitute a significant proportion of the critical healthcare workforce (such as part-time workers, nurses, ancillary workers and those with caring responsibilities). However, analysis of persuadability scores and the acceptability of individual scenarios on the basis of HCWs' demographic and employment characteristics showed that the groups who may be most in *need *of suitable interventions to persuade them to attend work may also be the least *receptive *to these interventions.

The findings from the qualitative phase of this study demonstrated that HCWs' decisions to work during pandemic influenza are affected by both barriers to *willingness *(for example, a perceived duty to care for a family member may make an HCW unwilling to work), and barriers to *ability *(such as being incapacitated by illness oneself, or unable to work because of disruption to transport and other services) [12]. The line between these barriers may be blurred (in the case of childcare obligations for example). Nevertheless, interventions to persuade otherwise reluctant HCWs to continue working may be more effective at overcoming barriers to *ability *(such as the provision of transport by employers to facilitate HCW attendance at work or the provision of childcare services if schools or nurseries are closed), than overcoming barriers to *willingness*, which it may be argued encompass a number of more intractable issues. These issues surround decisions about the acceptability of risk [17]. Although changes to working conditions and the introduction of greater flexibilities may lessen absenteeism in theory by reducing the (statistical) risk of infection; in reality, the same interventions may still be deemed unacceptable by a large proportion of HCWs for whom the reduction in statistical risk is not accompanied by a commensurate increase in perceived risk *acceptability*.

## Limitations

There were some limitations to this study. First, the survey asked respondents to consider their potential response to a hypothetical situation. In an actual influenza pandemic, decisions by individual HCWs about whether to work, and the factors determining these decisions, may differ from those reported in response to the survey. Consequently, the likelihood and persuadability scores calculated for survey respondents cannot be considered a prediction of the percentage of HCWs who may or may not work during a pandemic, because we are unable to say how likely any of the stated scenarios (closure of schools etc.) are to arise. Nevertheless, these scores do give an indication of the factors that may increase or reduce HCWs' willingness to work, and the sorts of interventions which may have the most significant impact in reducing potential levels of absenteeism amongst HCWs during a pandemic.

Second, discrepancies in estimates of potential absenteeism rates amongst HCWs have been observed between different studies and in different geographical areas [1,3,4]. It is not possible to confirm whether these differences relate to the point in time at which individuals were surveyed, regional variations, or the methodology used to elicit information. Similarly, despite our analysis showing no evidence of non-response bias on the basis of either age or gender, the demographic similarity between respondents and non-respondents does not necessarily mean that these groups would be similar in their work-related behaviour during an influenza pandemic. Willingness to participate in the survey may be positively correlated with willingness to work, meaning that survey results may *under-estimate *potential absenteeism.

Finally, the survey response rate (34.4%, n = 1032) was relatively low in comparison to other postal surveys of healthcare professionals [18,19]. However, the number of responses received constitutes a larger sample size than other studies which have surveyed HCW response to extreme scenarios in general [1,20], and pandemic influenza in particular [3,4,14]. Many of these studies were conducted internationally, whereas the current research reports findings of direct relevance in shaping an evidence base for pandemic influenza planning in the UK context.

## Conclusion

Pandemic influenza is considered to be the most extreme risk to national security in the UK, and it is essential that in the event of a pandemic, health services are able to manage the severe demands that will be placed upon them. This research provides current information about the attitudes and needs of HCWs who would be at the front line of any response to pandemic influenza in the UK. Results from this study suggest that absenteeism amongst HCWs at any one time during an influenza pandemic may be as high as 85%, with potential absence from work particularly concentrated amongst nursing and ancillary workers. Although interventions designed to minimise absenteeism should target HCWs with a lower stated likelihood of working, members of these groups may also be the least receptive to such interventions. Changes to working conditions which reduce barriers to the *ability *to work may not address barriers linked to *willingness *to work, and may fail to overcome HCWs' reluctance to work in the face of what may be still deemed unacceptable risk to self and/or family. Future initiatives to reduce potential absenteeism amongst HCWs during an influenza pandemic should aim to identify interventions that will influence willingness to work rather than focusing simply on ability to work.

## Competing interests

The authors declare that they have no competing interests.

## Authors' contributions

Survey distribution and data collection were carried out by SD and JI, with analysis and interpretation carried out by SD in consultation with SW. The survey tool was designed by JI, HD, SW, SG, JP, JP and CG. The first draft of this article was composed by SD and was revised critically by all authors. All authors have approved the final version of the manuscript.

## Pre-publication history

The pre-publication history for this paper can be accessed here:



## Supplementary Material

Additional file 1 **Healthcare workers attitude questionnaire**. Health care workers attitude towards working during pandemic influenza. Click here for file
