# Supplementary material for: Will the NHS continue to function in an influenza pandemic? a survey of healthcare workers in the West Midlands, UK
Source: BMC Public Health. 2009 May 14;9:142. doi: 10.1186/1471-2458-9-142 (PMC2690584; doi:10.1186/1471-2458-9-142)
Supplement: Additional file 1 — Healthcare workers attitude questionnaire. Health care workers attitude towards working during pandemic influenza. [file 1471-2458-9-142-S1.doc]

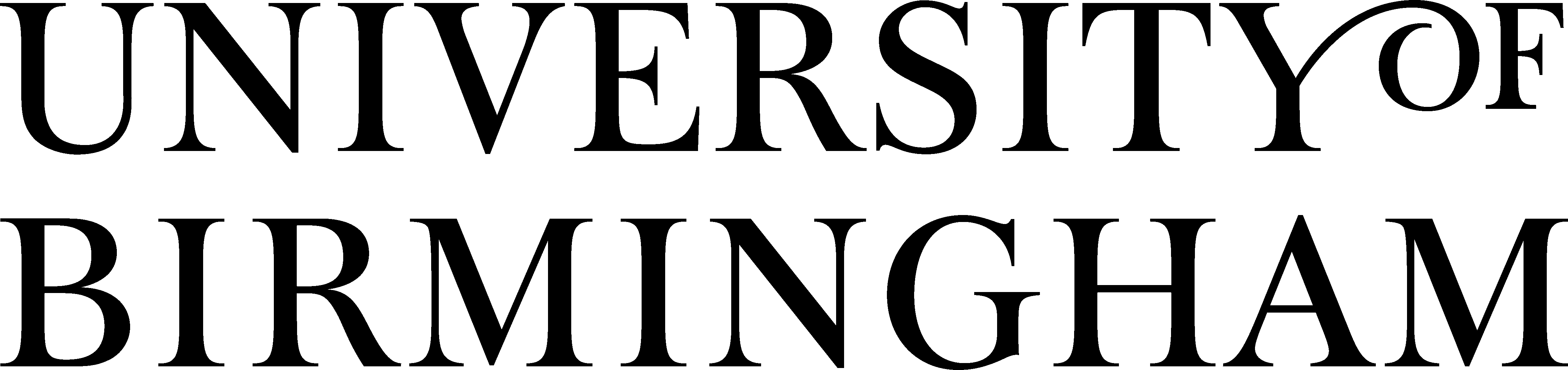
Principal Investigator: Dr Heather Draper

Research Fellow: Dr Jonathan Ives

Centre for Biomedical Ethics

Dept. of Primary Care and General Practice


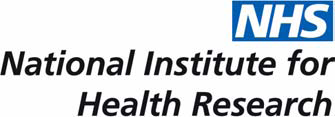
The University of Birmingham

Edgbaston

Birmingham

B15 2TT

T: 0121 415 8102

M: 07866 459 776

E: j.c.ives@bham.ac.uk

| **Health care workers’ attitudes to working during pandemic influenza** |
| --- |
| This questionnaire is about your attitudes to working during an influenza pandemic. An influenza pandemic is a worldwide outbreak of influenza that affects a very high proportion of the population. Your answers to these questions are very important to us and may help to inform UK emergency planning. All responses will be handled in total confidence and your answers will only be seen by the research team. ***No access*** *to individual questionnaires will be given to managers or others in your Trust/Practice*.  **This questionnaire should take no longer than 15 minutes to complete.**  If you have any questions about this questionnaire please contact Dr Jonathan Ives (see above for contact details)  **If you would prefer to complete this questionnaire online you may do so using the following weblink:**  **http://**[**www.pandemicflu.bham.ac.uk/Questionnaire_1.shtml**](http://www.pandemicflu.bham.ac.uk/Questionnaire_1.shtml)  **If you choose to complete this questionnaire online, you will need to enter the**  **six-digit number that is found in the bottom right hand corner of these pages.** |

| Please shade the circles in Blue or Black ink – NOT PENCIL  **Section One – what you believe about pandemic influenza** | | | | | | | | | | | | | | |
| --- | --- | --- | --- | --- | --- | --- | --- | --- | --- | --- | --- | --- | --- | --- |
| In this section we ask a few questions about pandemic influenza itself. This is not a test, and there are no right or wrong answers to these questions. Even the experts disagree on what the answers are. Your answers to these questions will allow us to better understand your responses to the rest of the survey. | | | | | | | | | | | | | | |
|  | | | | | | | | | | | | | | |
| **Q1.** | | | | | | | | | | | | | | |
|  | | | | | | | | **Yes** | | **No** | | | **Don’t know** | |
| (a) Do you think an influenza pandemic is going to happen? | | | | | | | | O | | O | | | O | |
| (b) Do you think that there will be a vaccine that can protect you against pandemic influenza? | | | | | | | | O | | O | | | O | |
| (c) Do you think that if there is an effective vaccine you will have access to it? | | | | | | | | O | | O | | | O | |
| (d) Do you think that everyone who catches pandemic influenza will recover from it? | | | | | | | | O | | O | | | O | |
| (e) Do you think that you will have access to protective equipment that will prevent you from catching pandemic influenza? | | | | | | | | O | | O | | | O | |
|  | | | | | | | | | | | | | | |
| **Q2.** | | | | | | | | | | | | | | |
|  | **60,000**  1/1000 people in the UK | | **600,000**  1/100 people  in the UK | | **1.5 million**  1/40 people  in the UK | **3 million**  1/20 people  in the UK | | | **6 million**  1/10 people  in the UK | | **15 million**  1/4 people  in the UK | | | **30 million**  1/2 people  in the UK |
| (a) In the UK, how many people do you think will CATCH pandemic influenza (there are 60 million people living in the UK)? | O | | O | | O | O | | | O | | O | | | O |
| (b) In the UK, how many people do you think will DIE FROM pandemic influenza (there are 60 million people living in the UK)? | O | | O | | O | O | | | O | | O | | | O |
|  | | | | | | | | | | | | | | |
| **Q3.** | | | | | | | | | | | | | | |
|  | | **Children** | | **Fit, healthy adults** | | | **The elderly** | | **Children AND fit, healthy adults** | | | **All at equal risk** | | |
| (a) Who do you think is most at risk of CATCHING pandemic influenza? | | O | | O | | | O | | O | | | O | | |
| (b) Who do you think is most at risk of DYING FROM pandemic influenza? | | O | | O | | | O | | O | | | O | | |

| **Section Two – attitudes to working during pandemic influenza** | | | | | | | |
| --- | --- | --- | --- | --- | --- | --- | --- |
| In this section we are asking you to tell us if you have any concerns about working during an influenza pandemic and in what circumstances you might be more or less likely to be willing to work.  We appreciate that these may be sensitive questions, and that you may feel that it is not appropriate to say that you would not come to work even if this is what you really feel. Please do not worry about anyone finding out about your answers – all these questionnaires are confidential and no-one, other than the research team, will see your answers. What is most important is that you tell us exactly how you feel and what you would do. It is likely that other people feel the same way as you. | | | | | | | |
| *Please shade the circle that most accurately represents your feelings. If the question is not applicable to you please shade ‘N/A’ (Not Applicable).* | | | | | | | |
| **Q4. If there was an outbreak of pandemic influenza how likely is it that you would work in the following circumstances?** | | | | | | | |
|  | **Likely** | | **Don’t know** | | **Unlikely** | | **N/A** |
| (a) If there was a greater than usual risk of becoming infected at work and falling ill yourself | O | | O | | O | | O |
| (b) If there was a greater than usual risk of infecting your family | O | | O | | O | | O |
| (c) If you were asked to take on different or additional work/duties for which you have not been trained | O | | O | | O | | O |
| (d) If you were asked to work more hours | O | | O | | O | | O |
| (e) If schools/nurseries were closed or other childcare services were disrupted | O | | O | | O | | O |
| (f) If your partner fell ill | O | | O | | O | | O |
| (g) If your children fell ill | O | | O | | O | | O |
| (h) If you were asked to work at a different hospital/healthcare practice to normal | O | | O | | O | | O |
| (i) If there was a shortage of fuel, leading to disruption of transport (private or public) | O | | O | | O | | O |
| (j) If you had to make decisions about who not to treat/care for | O | | O | | O | | O |
| (k) If you had to work with untrained volunteers or workers brought out of retirement | O | | O | | O | | O |
| (l) If your colleagues were dying | O | | O | | O | | O |
| *Please shade the circle that most accurately represents your feelings. If the question is not applicable to you please shade ‘N/A’ (Not Applicable):* | | | | | | | |
| **Q5. If there was an outbreak of pandemic influenza would you be MORE likely to work if:** | | | | | | | |
|  | | **More likely** | | **About the same** | | **N/A** | |
| (a) You were allowed to work at the nearest hospital/healthcare practice to your home | | O | | O | | O | |
| (b) You were provided with accommodation so that you do not take the infection home | | O | | O | | O | |
| (c) Your employer provided transport for you to get to work and home again | | O | | O | | O | |
| (d) Childcare was provided for you | | O | | O | | O | |
| (e) You were offered vaccination (if available) and/or offered treatment if you fell ill | | O | | O | | O | |
| (f) Your family were offered vaccination (if available) and/or offered treatment if they fell ill | | O | | O | | O | |
| (g) Your employer shared their emergency plans with you, and told you in advance what would be expected of you during an influenza pandemic | | O | | O | | O | |
| (h) You were offered personal protective equipment when working with or around affected patients | | O | | O | | O | |
| (i) Your employers accepted liability for any mistake you make whilst doing a job you are not trained for so that you could not be sued | | O | | O | | O | |
| (j) You were allowed to work more flexible hours | | O | | O | | O | |
| (k) You were paid a top-up salary that was appropriate to the level of duties you were asked to take on during an influenza pandemic | | O | | O | | O | |
| (l) You were provided with life/disability insurance | | O | | O | | O | |
| (m) Is there anything we have not mentioned that would make you more or less likely to go to work during an influenza pandemic?  If so please specify: …………………………………………………………………………………………………………………………... | | | | | | | |

| *Please shade the circle that most accurately represents your feelings. If the question is not applicable to you please shade ‘N/A’ (Not Applicable):* | | |
| --- | --- | --- |
| **Q6. Please indicate whether you tend agree or disagree with the statements below.** | | |
|  | **Tend to agree** | **Tend to disagree** |
| (a) Doctors and nurses have a duty to tend the sick even when there are high risks to themselves or their families | O | O |
| (b) Healthcare workers should **not** be given any special priority during an influenza pandemic and everyone should have equal access to treatment | O | O |
| (c) Everyone working in the health service, not just doctors and nurses, has a duty to work during a health emergency, even if there are high risks involved | O | O |
| (d) Healthcare workers should lose wages if they are unwilling to work during an influenza pandemicealthcareH | O | O |
| (e) People should do what their religious leaders advise or what their faith directs | O | O |
| (f) Everyone should pull together during an influenza pandemic | O | O |
| (g) Healthcare workers should be allowed to refuse to work with, or near, infected patients | O | O |
| (h) Professional bodies and unions should offer explicit guidance about whether or not there is a duty to work during an influenza pandemic | O | O |
| (i) People who refuse to work in a time of national crisis should be punished in some way | O | O |
| (j) People who work through a national crisis should be rewarded in some way | O | O |
| (k) My main responsibility is to myself and my family and my family will take priority over my work | O | O |
| (l) I have to go to work because I couldn’t manage if I lost any of my wages | O | O |
| (m) My employer has a responsibility to offer protective equipment if I am asked to work during an influenza pandemic | O | O |
| (n) My employer has a responsibility to offer me a vaccination, if one becomes available, if I am asked to work during an influenza pandemic | O | O |
| (o) My employer has a responsibility to offer my family a vaccination, if one becomes available, if I am asked to work during an influenza pandemic | O | O |
| (p) Healthcare workers should face disciplinary action if they are unwilling to work during an influenza pandemic | O | O |

| **Section Three – information about you** | | | | | | | | | | | | | | | | | |
| --- | --- | --- | --- | --- | --- | --- | --- | --- | --- | --- | --- | --- | --- | --- | --- | --- | --- |
| In this section we are asking a series of questions that may appear to be quite personal. However, the reason for asking these questions is for us to get a better understanding about which groups of people have different views. Your answers in this section are very important to us, because they will enable us to better understand if some groups of people (such as those who have young children or are carers for other relatives or have older dependents etc.) tend to have different views to other groups of people. This information will not be used to identify you and all reports written about this study will ensure that you cannot be identified by the answers you give. If there is any question that you feel uncomfortable answering please leave it blank and go on to the next question. | | | | | | | | | | | | | | | | | |
|  | | | | | | | | | | | | | | | | | |
| **Q7. Gender** | Male | | O | | |  | **Q8. What is your country of birth?** | | | | | | | | | | |
|  | Female | | O | | |  | ………………………………………… | | | | | | | | | | |
|  | | | | | | |
|  | | | | | | | | | | | | | | | | | |
| **Q9. What is your age group?** | | | | | | | | | | | | | | | | | |
|  | | | | | | | | | | | | | | | | | |
| 16 - 20 years | O | | | | 21 - 30 years | | | O | | | | | | 31 - 40 years | | O | |
|  | | | | | | | | | | | | | | | | | |
| 41 - 50 years | O | | | | 51 - 60 years | | | O | | | | | | 61 years or more | | O | |
|  | | | | | | | | | | | | | | | | | |
| **Q10. Please indicate which of these best describes your living arrangements (you can mark more than one)** | | | | | | | | | | | | | | | | | |
|  | | | | | | | | | | | | | | | | | |
| Live with spouse/partner and children/grandchildren under 16 | | | | | | | | | O | |  | Live alone | | | | | O |
| Live with spouse/partner, no children/grandchildren under 16 | | | | | | | | | O | |  | Share with friends | | | | | O |
| Single parent/grandparent with children under 16 at home | | | | | | | | | O | |  | Share with parents/other relatives | | | | | O |
| Single parent, no children under 16 at home | | | | | | | | | O | |  |  | | | | |  |
|  | | | | | | | | | | | | | | | | | |
| **Q11. Do you have any elderly dependents?** | | | | | | | | | | | | |  | **Q11a. If you answered ‘yes’ to question 11, do you consider yourself to be a carer?** | | | |
|  | | | | | | | | | | | | |  |  | | | |
| No | | O | | Yes, living close to me | | | | | | O | | |  | Yes | O | | |
| Yes, living with me | | O | | Yes, living distant to me | | | | | | O | | |  | No | O | | |

| **Section Four – information about your job** | | | | | | | | | | | | | | |
| --- | --- | --- | --- | --- | --- | --- | --- | --- | --- | --- | --- | --- | --- | --- |
| In this section we are asking a series of questions about your job. Your answers to these questions are important to us because they will allow us to get a better understanding of the views that different sorts of people have. | | | | | | | | | | | | | | |
| **Q12. Please tell us about your role in healthcare (please answer a, b and c as appropriate)** | | | | | | | | | | | | | | |
|  | | | | | | | | | | | | | | |
| (a) What is your occupation (i.e. nurse, pharmacist, porter, doctor, phlebotomist etc.)?  ……………………………………………………………………………………….. | | | | | | | | | | | | | | |
| (b) What is your speciality, if applicable (i.e. critical care, neurology, cardiology, A&E, paediatrics etc.)?  ……………………………………………………………………………………….. | | | | | | | | | | | | | | |
| (c) In what kind of healthcare setting do you work (i.e. district general hospital inpatient/outpatient, community hospital, primary care, private sector etc.)?  ……………………………………………………………………………………….. | | | | | | | | | | | | | | |
|  | | | | | | | | | | | | | | |
| **Q13. Please provide information on the nature of your employment (please answer a, b, c and d. Are you:** | | | | | | | | | | | | | | |
|  | | | | | | | | | | | | | | |
| (a) | Full-time? | | | O | | |  | | | (b) | | A line manager? | | O |
|  | Part-time? | | | O | | |  |  |  |  |  | Not a line manager? | | O |
|  | | | | | | | | | | | | | | |
| (c) | A shift worker? | | | O | | |  | | | (d) | | An agency/bank worker? | | O |
|  | Not a shift worker? | | | O | | |  |  |  |  |  | Employed directly by Hospital/Practice? | | O |
|  |  | | |  | | |  | | |  | |  | |  |
| **Q14. For how long have you been working in a healthcare setting?** | | | | | | |  | **Q15. For how long have you been working for your current employer?** | | | | | | |
| ………...........years……………months | | | | | | |  | ………...........years……………months | | | | | | |
|  | | | | | | | | | | | | | | |
| **Q16. How long, on a typical day, does it take you to travel from home to work and back again?** | | | | | | | | | | | | | | |
| …………..hours……….….minutes | | | | | | | | | | | | | | |
|  | | | | | | | | | | | | | | |
| **Q17. How do you usually travel to and from work? (you can shade more than one)** | | | | | | | | | | | | | | |
| Private car | | O | Walk/cycle | | O | Public transport (bus, train, etc.) | | | O | | Other (please specify | | O  …………………. | |

| **Q18a. Are there any concerns you have that might be specific to your culture or religion that we have not explored in this questionnaire? Please tell us if we have missed out anything that you think is important.** |
| --- |
| …………………………………………………………………………………………………..............................................................  …………………………………………………………………………………………………..............................................................  ………………………………………………………………………………………………….............................................................. |
|  |
| **Q18b. If you would like to make any other comments, please do so in the space provided below. You do not have to write anything.** |
| …………………………………………………………………………………………………..............................................................  …………………………………………………………………………………………………..............................................................  ………………………………………………………………………………………………….............................................................. |

**Thank you for taking the time to complete this questionnaire.**

**Your answers are very important to us, and will be treated in complete confidence.**

**Please return your completed questionnaire to Dr Jonathan Ives in the pre-paid envelope provided.**
